# The long‐term effects of a school‐based intervention on preventing childhood overweight: Propensity score matching analysis within the Generation R Study cohort

**DOI:** 10.1111/ijpo.13200

**Published:** 2025-01-06

**Authors:** Famke J. M. Mölenberg, Michel S. Smit, Daan Nieboer, Trudy Voortman, Wilma Jansen

**Affiliations:** ^1^ Department of Public Health, Erasmus MC University Medical Centre Rotterdam Rotterdam the Netherlands; ^2^ The Generation R Study Group, Erasmus MC University Medical Centre Rotterdam Rotterdam the Netherlands; ^3^ Department of Epidemiology, Erasmus MC University Medical Centre Rotterdam Rotterdam the Netherlands; ^4^ Department of Social Development The City of Rotterdam Rotterdam the Netherlands

**Keywords:** children, exercise, healthy lifestyle, overweight, school intervention

## Abstract

**Background:**

This study investigated the long‐term impact of the primary school‐based multicomponent lifestyle intervention “Lekker Fit!” (LF) on obesity‐related outcomes, and studied whether the impact differed between population subgroups.

**Methods:**

Children from the Generation R Study (Rotterdam, the Netherlands) were categorized into the LF group (6 years exposure, between the ages 6/7 to 12/13 years) or regular school group (no exposure). BMI and DXA‐derived fat mass were assessed after 4 years of intervention (age 10 years), and 1.5 years post‐intervention (age 14 years). A propensity score matching model was fitted to examine the intervention effect on BMI‐z‐score and percent fat mass, and we tested for differences by sex, pre‐intervention weight status, ethnic background, and income.

**Results:**

We found no effect on BMI‐z‐score [0.06 (95% confidence interval [CI]: −0.04 to 0.17)] and percent fat mass (0.4%‐point [95% CI: −0.2 to 1.1]) after 4 years of intervention. 1.5 years post‐intervention and after 6 years of exposure, BMI‐z‐score (0.11 [95% CI: 0.00–0.22]) and percent fat mass (1.1%‐point [95% CI: 0.2–1.9]) were significantly higher for children in the LF group. No subgroup differences were found.

**Conclusion:**

Findings suggest the need for obesity prevention programs that extend beyond primary education.

## 1 INTRODUCTION

Childhood overweight and obesity is one of the larger public health crises we are currently facing.^1^, ^2^, ^3^ School‐based interventions with diet and physical activity (PA) components hold promise for childhood obesity prevention,^4^, ^5^, ^6^, ^7^ but evidence for the long‐term effects of these interventions is lacking.^4^, ^5^, ^6^, ^7^, ^8^, ^9^

Classically, randomized controlled trials (RCTs) are considered the golden standard to estimate the effect of interventions.^10^ However, RCTs have some drawbacks when studying school‐based interventions, such as the challenges in capturing long‐term effects and the difficulty in conducting adequately powered subgroup analyses to study potential differential effects.^4^ Moreover, it might be ethically challenging to prohibit control schools from implementing health promotion interventions if favourable short‐term effects are observed. Practice‐based evidence could offer valuable insights into the effectiveness of health promotion activities.^11^, ^12^, ^13^

One example of a primary school‐based multicomponent lifestyle intervention for which short‐term benefits are found, but long‐term impacts remain unknown, is “Lekker Fit!” (LF; which translates to “Enjoy being fit!”).^14^, ^15^ The intervention, implemented in the city of Rotterdam, the Netherlands, specifically targets primary schools where children are at the highest risk of becoming overweight. The results of the RCT played a significant role in the decision to implement the intervention in the following years, leading to its coverage in nearly half of the primary schools in the city.

In this study, we used data from a birth‐cohort study to evaluate the long‐term effects of this intervention. The selective exposure of children to the intervention has severe implications for causal inference, since there are many factors why children from targeted schools may have higher overweight and obesity rates. These factors include, but are not limited to poverty, health literacy of the parents, and access to care. In this study, detailed data of the children and their families were collected before the intervention started and therefore we used matching to make groups comparable. The objective of this study was to evaluate the long‐term effect of the intervention on BMI and fat mass, and to explore whether the intervention effects differed for population subgroups.

## 2 METHODS

### 2.1 Study context

The city of Rotterdam is the second largest city of the Netherlands and hosts a multi‐ethnic and multicultural population. Figure S1, Supporting Information visualizes the percentage of children with overweight in the city during childhood, collected by the local Child Health Centres (2015–2019). The majority of LF intervention schools (73%) were located in five areas of the city. Overweight percentages were presented for children living in areas primarily targeted by the intervention (*n* = 5) and other areas (*n* = 6). Overweight prevalence was 15.3% and 12.2% for 5/6 year olds, 27.9% and 21.8% for 9/10 year olds, and 27.4% and 22.5% for 13/14 year olds, respectively, for children living in target and non‐target areas.

### 2.2 Study design

**FIGURE 1:**
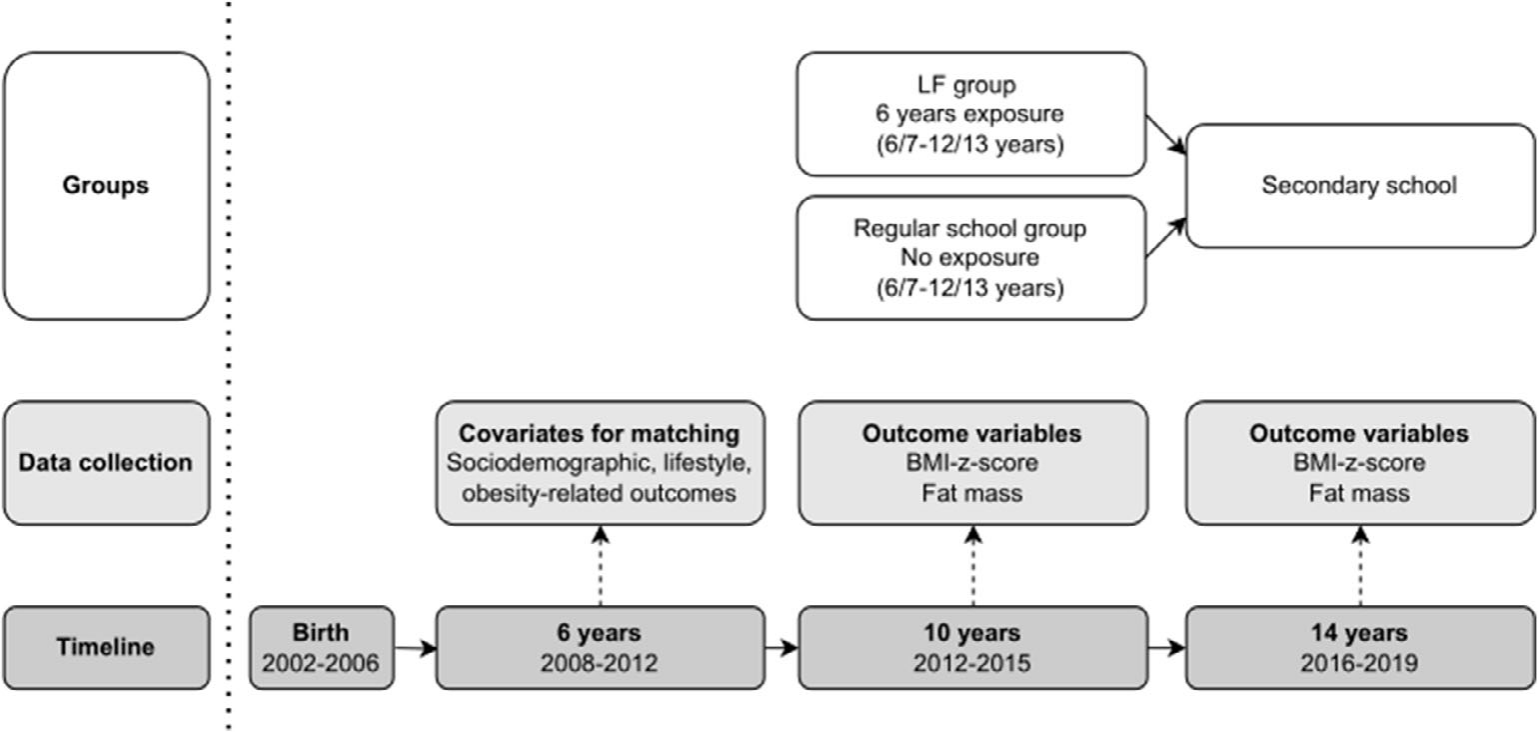
Study design to evaluate the long‐term impact of “Lekker Fit.”

The study design is visualized in Figure 1. We used data from the Generation R Study, an ongoing birth‐cohort study. All pregnant women who had an expected delivery date between April 2002 and January 2006 and who lived in the city of Rotterdam, the Netherlands, were invited to participate and children and their parents were followed over time. The RCT took place in 2006/2007 and focused on 6–12‐year‐old children. It is unlikely that children in the Generation R Study were participants of the RCT. More information is presented in the design and cohort update paper.^16^ The Medical Ethics Committee of Erasmus University Medical Centre Rotterdam approved the study (MEC 217.595/2002/20).

For this study, we used information collected at the age of 6, 10, and 14 years. Written informed consent was obtained from parents until the child was 10 years, and from parents and children at the age of 14 years.

### 2.3 The Lekker Fit! intervention

The LF intervention targets primary schools located in socioeconomically disadvantaged neighbourhoods, which host a population at the highest risk of developing overweight and obesity. The intervention aims at promoting healthy eating and active living by changing individual behaviours of children and their parents, as well as school policies and the school curriculum. Main component of the intervention is that children receive three physical education (PE) sessions a week provided by a professional PE teacher, whereas two PE classes a week by a classroom teacher constitute the usual mandatory curriculum. The intervention started in 2006/2007, and additional intervention components were added to the intervention in more recent years. Details about the intervention are presented in Table 1 and can be found elsewhere.^17^ The number of schools participating in the intervention increased from 20 in 2006 to 94 in 2020, reaching 18 thousand children annually.^18^

**TABLE 1:** Description of the intervention.

Description	
Initial intervention components tested during the RCT in 2006/2007^14^, ^15^	
1	Three PE sessions a week provided by a trained PE teacher to children of grade 3–8 (6/7–12/13 years)
2	Organization of additional sport and play activities outside school hours
3	Three lessons per week of classroom education provided by the classroom teacher. Themes of the classes are healthy nutrition, active living, and healthy lifestyle choices
4	Annual height and weight measurements, and fit‐test conducted by the PE teacher. Results are communicated to the children and parents, and individual counselling by the school nurse is offered
5	Health promotion gathering at the beginning of the school year for parents, and the involvement of local sport clubs in providing some of the PE classes and sport and play activities outside school hours
Additional intervention components added to the intervention in more recent years	
6	Since 2012: Expansion of the intervention to children of grade 1 and 2 (4–6 years)
7	Since 2013: Implementation of the water campaign; children drink water at least two times a day during school hours
8	Since 2013: Implementation of the “enjoy fruit” component; children only eat fruit or vegetables during their morning break
9	Since 2015: Implementation of the “treats” component; guidelines for in‐class treats shared during birthday festivities

### 2.4 Study population

For this study, we linked cohort data to municipal registry databases to obtain information on the school careers of the children. Gender of the child, and the day of birth of the child and mother were used for linkage. Linkage was approved by privacy officers from the City of Rotterdam and Erasmus Medical Centre Rotterdam.

**FIGURE 2:**
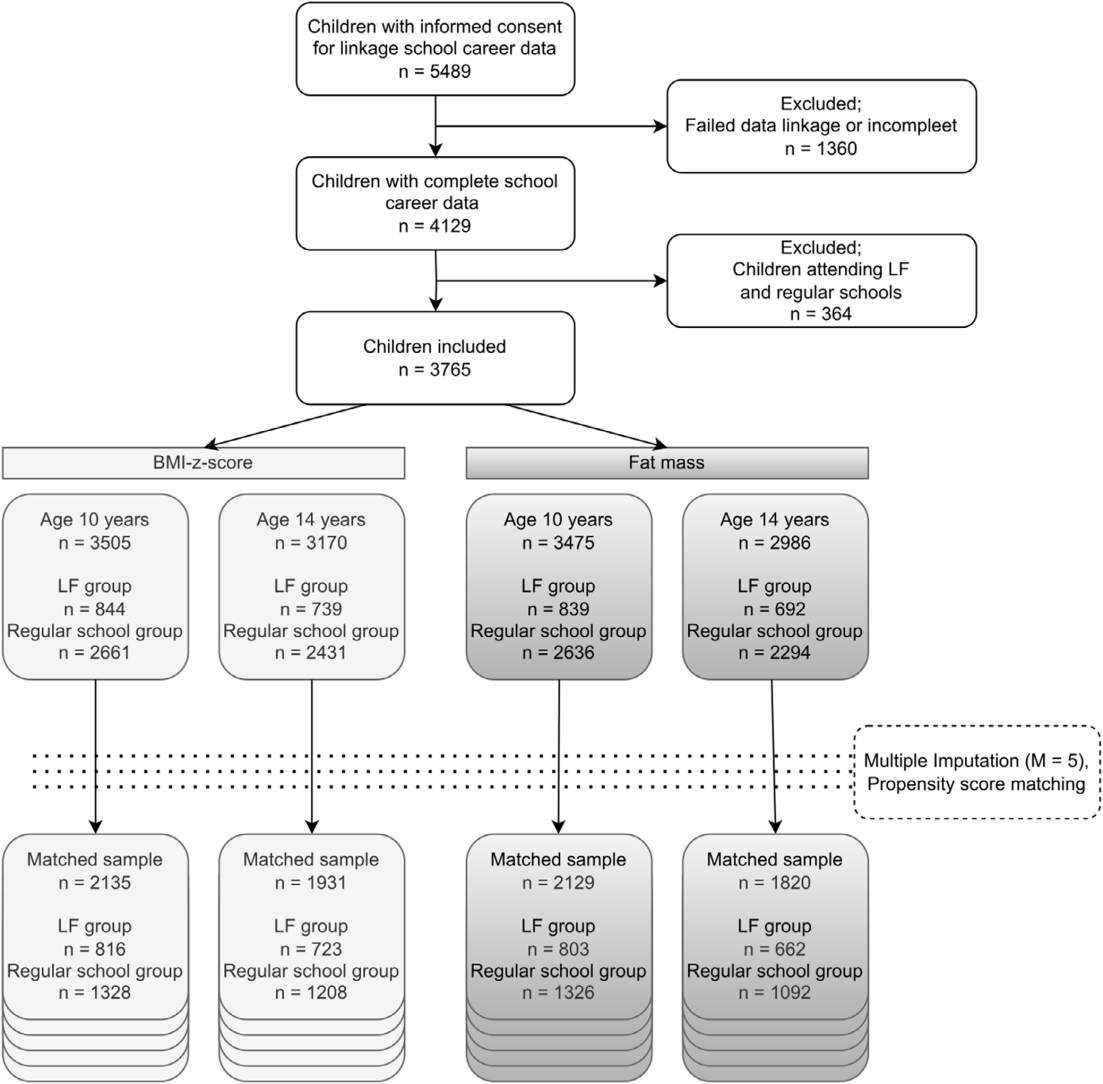
Flow diagram.

In total, 9901 children and their parents participated in the Generation R Study at baseline, and 5489 parents gave consent for linkage with school data from municipal registration databases. We excluded children for who linkage was not possible (*n* = 1316) and without information on the schools attended during the intervention period (*n* = 44). In total, 4129 children were eligible for the present study (Figure 2).

### 2.5 Exposure to the intervention

Primary school in the Netherlands is intended for children aged 4–12 years old and contains eight grades. Usually children transit to secondary school at the age of 12/13 years. From the municipal registration database we obtained, for each child, information on all primary schools attended. Date of entry and exit of schools were available, as well as a unique identifier per school. The database included children who were at least once registered at a school in the Rotterdam region. For each school, we received information on the yearly implementation status of the Lekker Fit intervention from the Department of Sport of the City of Rotterdam. This allowed to identify children who were exposed, and the years of exposure to the intervention from grades three to eight. Children were categorized into the LF group (6 years of intervention; *n* = 897), regular school group (≤6 months intervention; *n* = 2868), and a mixed group (some years of intervention; *n* = 364). The mixed group consisted of children attending a school that implemented the intervention during their school‐career, and children who changed schools. Given the small sample size, the mixed group were not used in analysis.

### 2.6 Obesity‐related outcomes

In the first 6 years since birth, body height and body weight measurements were performed during routine visits at the Child Health Centres. At the age of 6, 10, and 14 years, children visited the research centre for detailed physical examinations. Body height and body weight were measured by trained staff during the research visit with the children not wearing shoes and heavy clothing. Body height was measured in standing position to the nearest 0.1 cm using a Harpenden stadiometer (Holtain Limited), and body weight was measured to the nearest 0.1 kg using a mechanical personal scale (SECA). BMI was calculated [weight (kg)/height (m^2^)] and age‐ and sex‐specific z‐scores for BMI were obtained from Dutch reference growth charts (Growth Analyzer 4.0, Dutch Growth Research Foundation).^19^ BMI was also categorized into normal weight, overweight, or obese, based on the cut‐offs published by the International Obesity Task Force.^20^

Body composition was measured at the research centre at the age of 6, 10, and 14 years using a DXA scanner (iDXA, GE‐Lunar, 2008, Madison, WI) using enCORE software version 12.6. Children were placed without shoes, heavy clothing, and metal objects in supine position on the DXA table. We calculated percent fat mass.

### 2.7 Covariates

Sociodemographic covariates were obtained at baseline of the Generation R Study by means of questionnaires and included age, sex and ethnic background, and net household income. Like in the RCT, a child's ethnic background was based on country of birth of the parents, and categorized into native Dutch, Turkish, Moroccan, Surinamese, Antillean, Cape Verdean, and other. Maternal country of birth was chosen when parents were born in different countries. For parental education level and net household income, we used information at baseline of the intervention (i.e., age 6 years). The highest education level attained of both parents was categorized according to the Dutch Standard Classification of Education into high (university degree), mid–high (higher vocational training, bachelor's degree), mid–low (>3 years general secondary school, intermediate vocational training), and low (no education, primary school, lower vocational training, intermediate general school, or ≤3 years general secondary school).^21^ Net household income was categorized into low (≤€2000/month), intermediate (<€2000–€3200/month), and high (>€3200/month).

In addition, we included six covariates to adjust for the potential confounding effect of health‐related behaviours at baseline of the intervention (i.e., age 6 years) on the development of overweight in children. We included the number of snacks consumed on a weekday and weekend day, and number of sugar‐sweetened beverages (SSB) consumed on a weekday and weekend day, frequency of outdoor play and sports participation as covariates. All were obtained from parent‐reported questionnaires at baseline of the intervention (i.e., age 6 years). Snack and SSB consumption were categorized into zero or one snack/SSB on a weekday/weekend day, and two or more snacks/SSB on a weekday/weekend day. Number of days playing outside was categorized into low (≤5 days/week) and high (>5 days/week). Sports participation was categorized into participating in any organized sport activity (yes, no).

### 2.8 Statistical analysis

Four separate study samples were constructed. In the first sample, we included children with BMI measured at age 10 to study the effect of the intervention on BMI‐z‐scores after 4 years of exposure to the intervention. In the second sample, we included children with BMI measured at age 14 to study the sustained effects on BMI‐z‐scores, 1.5 years after transitioning from primary to secondary school and after being exposed for 6 years to the intervention. The same was done for percent fat mass, resulting in four study samples. Multiple imputation by chained equations was used to impute missing data on covariates in each sample (*M* = 5). Table S1 presents the number of missings per variable.

We used propensity score matching to identify a regular school group that was similar to the children in the LF group. In sufficiently powered RCTs, random allocation of schools to the intervention or control group will ensure that important covariates are balanced over the two groups. Propensity score matching can be used to make groups comparable in situations for which it is not ethically or feasible to randomize.^22^, ^23^ This methodology includes two steps. First, the propensity of being exposed to the intervention is estimated for each child, based on observed characteristics. Second, the estimated propensity score, ranging between 0 and 1, is used to match children with similar scores from the intervention and control group. Propensity score matching assumes that, conditional on the included observed characteristics, children in the regular school group are similar to children in the LF group.

Separate logistic regression models were fitted, where treatment status was regressed on baseline covariates when the children were 6 years old. Potential confounders included were age, sex, ethnicity, maternal education level, paternal education level, net household income, weekday and weekend day snack consumption, weekday and weekend day SSB consumption, outdoor play, sports participation, BMI‐z‐score, percent fat mass, and weight status. We also added the child‐specific slope for the trend in BMI‐z‐scores using information retrieved from the Child Health Centres collected in the first 6 years of life. Trend in BMI‐z‐score was obtained from a linear mixed‐effects model that included child‐specific slopes and intercepts, and the covariate ethnicity. The estimated propensity scores were used to match the children. We ran several matching methods (1:1, 1:2, and 1:3 nearest neighbour matching without replacement, and full matching estimated with probit regression). We initially set the calliper width at 0.1, and checked the number of successful matches and covariate balance between children in the matched sample by means of visual inspections and standardized mean differences. Best matching was achieved when using 2:1 nearest neighbour matching without replacement, and these findings were reported.

We checked if the matching resulted in groups that are similar. We plotted the distribution of propensity scores in the intervention and regular school group to evaluate if estimated scores sufficiently overlap. Furthermore, we calculated the standardized mean difference (SMD) to examine the balance of covariate distribution between the two groups before and after matching. Smaller SMDs indicates that groups are more comparable.

For each analytical sample, we described baseline characteristics by treatment group when the children were 6 years old. We estimated the causal average effect of the intervention by performing regression analyses using BMI‐z‐score and percent fat mass as outcomes and using the intervention status as a covariate. The intervention effect, comparing outcomes between children exposed to the intervention to children without intervention, was estimated using *t* tests. The analyses were performed on each of the imputed datasets and results were subsequently pooled using Rubin rules.

Children attending the same schools tend to be more similar than those attending other schools, and we carefully examined whether clustering at the school‐level was of importance in our sample. In our final sample many schools were attended by only few children (median 4 children per school; IQR [1–10]), and therefore we did not take clustering into account at the level of schools.

#### 2.8.1 Subgroup analysis

In line with the RCT conducted in 2006/2007,^15^ we evaluated differences by sex and pre‐intervention weight status. The intervention target deprived neighbourhoods, which host relatively more persons from lower socioeconomic and ethnic minority groups. To understand for who the intervention is more beneficial, we additionally explored whether the observed effects differed by the child's ethnicity and household income. To retain statistical power, we categorized ethnic background into native‐Dutch and non‐native Dutch. Net household income was dichotomised into higher (>€3200/month) and lower than average Dutch net household income (≤€3200/month). We conducted stratified analysis and tested for differences using *Z* tests.

#### 2.8.2 Sensitivity analysis

For the estimation of the intervention effect after transitioning to secondary school and after the intervention ended, we excluded children <1 year since primary school. This to avoid that the estimated treatment effect was attributable to the children who were relatively recently exposed to the intervention.

All analyses were conducted in R version 3.4.1, using the mice package for multiple imputation and the MatchThem package for the propensity score matching. Two‐sided *p*‐values <0.05 were considered statistically significant.

## 3 RESULTS

### 3.1 Description of the analytical samples

In all analytical samples, we observed a substantial overlap of the distribution of the propensity scores between the intervention and regular school group, and matching reduced the absolute standardized differences of the means for all covariates were below 0.1 (Figures S2–S9). The size of the matched sample ranged between 1820 for percent fat mass at age 14 years, and 2135 for BMI‐z‐score at age 10 years.

Table 2 presents the baseline characteristics of the children before matching and in the propensity score matched sample for children with BMI at age 10. After matching, covariates were well balanced between the two groups. Children in the matched sample were on average 6.1 years old at baseline, and 51.9% were girls. Most children were of non‐native Dutch ethnicity (57.5%). About half of the children had parents with high or mid–high education level, and a third had a net household income <€2000/month. Mean BMI‐z‐score was 0.30 and the prevalence of overweight was 22.0%. Mean percent fat mass was 28.3%.

**TABLE 2:** Baseline characteristics of the children before matching (*n* = 3505) and in the propensity score‐matched sample (*n* = 2135) for BMI at age 10 years old.

	Before matching^a^		After matching^b^	
	Regular school group (*n* = 2661)	LF group (*n* = 844)	Regular school group (*n* = 1322)	LF group (*n* = 813)
Age (mean in years)	6.1	6.2	6.1	6.1
Girls (%)	50.4	51.5	52.2	51.3
Ethnicity (%)				
Native‐Dutch	69.1	41.4	42.5	42.6
Turkish	2.9	10.1	8.1	9.0
Moroccan	2.8	10.4	8.4	9.1
Surinamese	6.2	12.4	12.9	12.4
Antillean	1.9	4.0	3.9	3.8
Cape Verdean	2.4	4.6	5.5	5.0
Other	14.6	17.0	18.6	18.1
Maternal education level (%)				
High	33.2	20.4	20.2	19.4
Mid–high	31.0	22.7	23.6	23.0
Mid–low	28.3	37.3	38.9	39.5
Low	7.5	19.6	17.3	18.1
Paternal education level (%)				
High	38.4	24.1	22.3	21.3
Mid–high	24.8	18.4	19.1	19.2
Mid–low	25.2	33.8	34.2	34.7
Low	11.6	23.6	24.5	24.9
Net household income (%)				
<€2000/month	15.2	32.4	33.8	34.7
€2000–€3200/month	23.6	35.1	36.1	34.9
>€3200/month	61.2	32.4	30.1	30.4
≥2 snacks on a weekday	26.6	30.1	28.8	29.4
≥2 snacks on a weekend day	51.9	50.7	51.1	50.9
≥2 SSBs on a weekday	69.2	71.6	71.0	71.3
≥2 SSBs on a weekend day	74.9	76.1	75.3	74.9
≤5 days/week playing outside (%)	31.2	39.9	38.9	41.0
No sport participation (%)	52.0	64.6	62.9	63.4
BMI‐z‐score (mean)	0.20	0.31	0.29	0.32
Overweight/obese (%)	14.0	22.1	21.5	22.8
Fat mass (mean percentage)	27.2	28.2	28.2	28.4

Abbreviations: BMI, body mass index; SSB, sugar‐sweetened beverage.

^a^ Non‐imputed sample, number of missing per variable are presented in Data S1.

^b^ With imputations.

### 3.2 Intervention effect on BMI and fat mass

Table 3 presents the estimated long‐term treatment effect of the intervention on BMI and fat mass. After 4 years of exposure, we found no intervention effects on BMI‐z‐score [0.06 (95% confidence interval [CI]: −0.04 to 0.17)] and percent fat mass (0.4%‐point [95% CI: −0.2 to 1.1]). 1.5 years after transitioning from primary to secondary school and after being exposed for 6 years to the intervention, BMI‐z‐score (0.11 [95% CI: 0.00–0.22]), and percent fat mass (1.1%‐point [95% CI: 0.2–1.9]) were significantly higher for children in the LF group.

**TABLE 3:** Long‐term treatment effect of the intervention on BMI and percent fat mass.

	BMI‐z‐score			Percent fat mass		
	LF group	Regular school group	Intervention effect (95% CI)	LF group	Regular school group	Intervention effect (95% CI)
After 4 years of exposure (age 9/10 years)	0.42	0.37	0.06 (−0.04 to 0.17)	31.4	30.9	0.4 (−0.2 to 1.1)
1.5 years post‐intervention, after 6 years exposure (age 14 years)	0.45	0.34	0.11 (0.00–0.22)	27.6	26.4	1.1 (0.2–1.9)

*Note*: Results are the estimated treatment effect for BMI‐z‐score and percent fat mass obtained from a linear regression model on a sample matched on propensity scores that included the covariates age, sex, ethnicity, maternal education level, paternal education level, household income, weekday snack consumption, weekend day snack consumption, weekday SSB consumption, weekend day SSB consumption, outdoor play, sports participation, BMI‐z‐score, percent fat mass, weight status, and trend in BMI‐z‐score.

Abbreviations: CI, confidence interval; BMI, body mass index; LF, Lekker Fit (Enjoy being fit!); SSB, sugar‐sweetened beverage.

### 3.3 Equity effect

We explored whether differential intervention effects were observed sex, pre‐intervention weight status, ethnic background, and socioeconomic groups. The results of the stratified analyses are presented in Table 4. No evidence for subgroup differences was found.

**TABLE 4:** Long‐term treatment effect of the intervention on BMI and percent fat mass, stratified by sex, pre‐intervention weight status, ethnic background, and income.

	BMI‐z‐score				Percent fat mass			
	4 years exposure	*p*‐value	1.5 post‐intervention, after 6 years exposure	*p*‐value	4 years exposure	*p*‐value	1.5 post‐intervention, after 6 years exposure	*p*‐value
Boys	−0.01 (−0.16 to 0.14)	0.36	0.08 (−0.14 to 0.30)	0.95	0.4 (−0.7 to 1.4)	0.38	1.1 (−0.3 to 2.5)	0.67
Girls	0.09 (−0.06 to 0.23)		0.09 (−0.10 to 0.28)		0.7 (−0.2 to 1.5)		0.9 (−0.3 to 2.0)	
Non‐overweight	0.08 (−0.02 to 0.19)	0.33	0.11 (−0.01 to 0.24)	0.72	0.8 (0.1–1.4)	0.16	1.1 (0.3–2.0)	0.85
Overweight/obese	−0.01 (−0.16 to 0.15)		0.07 (−0.12 to 0.26)		−0.2 (−1.5 to 1.0)		1.3 (−0.8 to 3.5)	
Native‐Dutch ethnicity	0.05 (−0.10 to 0.19)	0.98	0.10 (−0.07 to 0.26)	0.98	0.4 (−0.6 to 1.4)	0.88	1.3 (0.1–2.5)	0.76
Non‐native Dutch ethnicity	0.05 (−0.09 to 0.18)		0.10 (−0.06 to 0.26)		0.5 (−0.4 to 1.4)		1.1 (−0.2 to 2.3)	
Income <€3200/month	−0.07 (−0.28 to 0.14)	0.18	−0.03 (−0.24 to 0.18)	0.34	−0.3 (−1.6 to 1.0)	0.13	0.7 (−0.7 to 2.0)	0.51
Income >€3200/month	0.10 (−0.05 to 0.25)		0.10 (−0.07 to 0.28)		0.9 (0.0–1.8)		1.3 (0.1–2.5)	

*Note*: Results are the estimated treatment effect for BMI‐z‐score and percent fat mass obtained from a linear regression model on a sample matched on propensity scores that included the covariates age, sex, ethnicity, maternal education level, paternal education level, household income, weekday snack consumption, weekend day snack consumption, weekday SSB consumption, weekend day SSB consumption, outdoor play, sports participation, BMI‐z‐score, percent fat mass, and trend in BMI‐z‐score.

Abbreviations: BMI, body mass index; CI, confidence interval; LF, Lekker Fit (Enjoy being fit!); SSB, sugar‐sweetened beverage.

### 3.4 Sensitivity analysis

Findings were not essentially different when excluding the children who were unexposed for less than 1 year. Higher BMI‐z‐score (0.12 [95% CI: −0.02 to 0.26]) and percent fat mass (1.1%‐point [95% CI: 0.1–2.1]) were seen for children from the LF group as compared to the regular school group.

## 4 DISCUSSION

This study evaluated the effectiveness of a school‐based multicomponent lifestyle intervention aimed at reducing overweight and obesity in children. The intervention reached approximately 18 thousand children residing in the city of Rotterdam on an annual basis. This study did not find significant intervention effects on BMI and fat mass after 4 years of exposure. After the transition from primary to secondary school and after a total of 6 years of exposure to the intervention, BMI and fat mass appeared to be higher for children who attended intervention schools. No subgroup differences were found.

A strength of this study was that we enriched a large database from a birth‐cohort study with municipal school data. We identified a cohort of approximately 800 children who were exposed to the intervention for 6 years, and used propensity score matching to identify a similar group of children attending schools that did not participate in the intervention. Children were matched using demographic, socioeconomic, lifestyle, and obesity‐related variables from before the intervention started. Outcome data were collected by trained researchers before, during, and after primary school, providing a detailed understanding of the development of BMI and percent fat mass during this crucial developmental period of life.

The main limitation of this study was uncertainty about the correct specification of the matching model. We included a wide variety of covariates and samples seemed well balanced after matching, but we cannot rule‐out the possibility that important unmeasured covariates have been missed. Furthermore, the covariates used for matching purposes were all derived from parental reports. The majority of items were unvalidated, and therefore it remains questionable if we measured what we intended to measure. We assume the use of unvalidated question items has a limited impact on our results, and do not expect systematic differences in reporting on these items between parents of children attending LF schools and their matched peers attending regular schools. The intervention primarily targets schools in socioeconomically disadvantaged neighbourhoods, and we matched children on various covariates, including the educational level attained by both parents and household income. Neighbourhood or institutional differences were not included in the matching procedure, and these factors might have introduced some confounding. We did not take clustering at the level of schools into account, because the majority of schools were attended by only few children. Some clustering may have been present, which may have resulted in smaller standard errors and narrow confidence intervals. Another limitation is that explorative analysis for differential effects had limited power. Ideally, we would have explored a dose–response relationship for the number of years being exposed to the intervention, but the group of children with some years of exposure was too small to derive meaningful conclusions.

In contrast to the earlier findings of a short‐term RCT with a 9‐month intervention duration, which revealed favourable effects on weight status, waist circumference, and physical test outcomes but not on BMI‐z‐scores, this current study did not find differences in BMI or fat‐mass after 4 years of intervention.^15^ Differences in timing, target population, study setup, and implementation may have contributed to the different findings. First, the RCT was conducted in the year 2006/2007, whereas in this study the majority of children were exposed to the intervention between 2010/2011 and 2016/2017. Over time, the Rotterdam environment became increasingly obesogenic, which may have reduced the effectiveness of the intervention.^24^ Second, differences in population characteristics became apparent. In the cluster RCT, 10.4% of the 6–9 year old children who participated had a native‐Dutch ethnic background, and the baseline prevalence of overweight was 24.4%.^15^ In the current evaluation, 42.6% of the children in the matched study sample had a native‐Dutch ethnic background, and 22.8% was overweight at the age of 6 years. These differences resulted from the expansion of the intervention at schools that hosted a different population, but also selective participation in the Generation R Study contributed to these differences. Third, randomisation ensured exchangeability between intervention and regular school groups, and it is essential to acknowledge that some confounding may have still been present in this study, particularly at the neighbourhood and institutional levels, even after matching the children for a wide variety of covariates. Fourth, the implementation of intervention components may have changed over time, or some components of the intervention or other lifestyle interventions may have been adapted at control schools. This may have resulted in comparing children following school programs that are less diverse than anticipated. Frequent interaction between policymakers and researchers could have been valuable in collecting all information needed—not only from intervention, but also from control schools—that would allow for better comparisons.

We conducted a comprehensive review of 19 studies examining the effects of school‐based obesity prevention interventions in children, when following children for at least 12 months post‐intervention.^9^ No clear evidence was found for long‐term effects on BMI, waist circumference, or weight status. We and other authors of reviews emphasized that only a handful of studies evaluated long‐term impacts, and higher quality follow‐up studies are needed.^4^, ^8^, ^9^ Following children in an experimental setting is often not possible, and exploring the possibilities of evaluating interventions through data already being collected may offer promising alternatives, especially when triangulated with findings from other data sources. The contexts in which interventions are implemented constantly change. Timely and regular interaction between policymakers, intervention developers, and researchers is crucial to ensure that interventions remain effective and relevant for their intended purposes. Furthermore, this interaction is needed to ensure no opportunities for meaningful evaluation are missed.^25^ Timely adapting interventions is recommended to ensure that interventions are aligned with current needs.

## 5 CONCLUSION

This study found no difference in obesity‐related outcomes after 4 years of LF intervention, a school‐based multicomponent lifestyle intervention introduced at primary schools in Rotterdam, the Netherlands. BMI and fat mass were higher for children from intervention schools 1.5 year after the intervention ended. To reduce overweight rates after the transition from primary to secondary education, sustained obesity prevention programs are required.

## AUTHOR CONTRIBUTIONS

FJMM, MSS, and WJ conceptualized and designed the study. FJMM, MSS, and DN performed data analysis. FJMM and MSS carried out the analysis. FJMM, MSS, DN, TV, and WJ interpreted the analysis. FJMM wrote the manuscript. MSS, DN, TV, and WJ contributed to the manuscript by critical revisions and giving comprehensive feedback on multiple drafts. FJMM, MSS, DN, TV, and WJ read and approved the final manuscript.

## CONFLICT OF INTEREST STATEMENT

No conflict of interest was declared.

## Supporting information


**Data S1.** Supporting Information.

## Data Availability

The dataset generated for the present study is not publicly available, as participants' consent was not obtained for data sharing. The Generation R Study has an open policy with regard to collaboration, and data are available for research groups when collaboration is established. More information is provided in the Generation R design paper.
