# Supplementary material for: The long‐term effects of a school‐based intervention on preventing childhood overweight: Propensity score matching analysis within the Generation R Study cohort
Source: Pediatr Obes. 2025 Jan 6;20(3):e13200. doi: 10.1111/ijpo.13200 (PMC11803176; doi:10.1111/ijpo.13200)
Supplement: Supplementary file 1 — Data S1. Supporting Information. [file IJPO-20-e13200-s001.pdf]

SUPPLEMENTAL MATERIALS BELONGING TO THE MANUSCRIPT

The long-term effects of a school-based intervention on preventing childhood overweight: propensity score matching analysis within the Generation R Study cohort

Famke J.M. Mölenberg[a,b], Michel S. Smit[a,b], Daan Nieboer[a], Trudy Voortman[c], Wilma Jansen[a,d]

[a] Department of Public Health, Erasmus MC, University Medical Centre Rotterdam, Rotterdam, The Netherlands

[b] The Generation R Study Group, Erasmus MC, University Medical Centre Rotterdam, Rotterdam, The Netherlands

[c] Department of Epidemiology, Erasmus MC, University Medical Center Rotterdam, Rotterdam, The Netherlands

[d] Department of Social Development, City of Rotterdam, Rotterdam, the Netherlands

Corresponding author

Famke J.M. Mölenberg, PhD, Department of Public Health, Erasmus MC, University Medical Centre Rotterdam, P.O. Box 2040, 3000 CA Rotterdam, The Netherlands. E-mail: f.molenberg@erasmusmc.nl

Supplemental Table 1: Number of missings per variable

	Missings (%)
Age	5.6
Gender	0.0
Ethnicity	2.0
Maternal education level	13.2
Paternal education level	20.5
Net household income	17.9
≥2 snacks on a weekday	13.8
≥2 snacks on a weekend day	13.8
≥2 SSBs on a weekday	13.9
≥2 SSBs on a weekend day	13.9
≤5 days/week playing outside	15.3
No sport participation	14.1
BMI-z-score	5.7
Overweight / obese	5.7
Fat mass	8.1

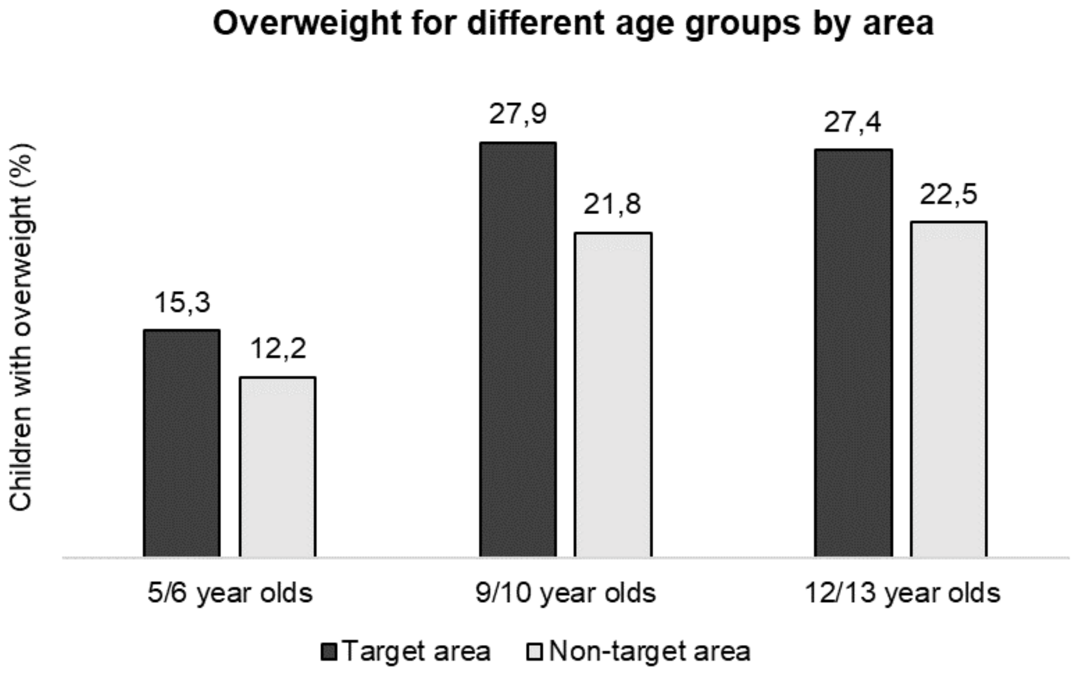

Supplemental Figure 1: Children with overweight for target (n=5) and non-target (n=6) area at different ages during childhood, using publicly available registry data (2015-2019)

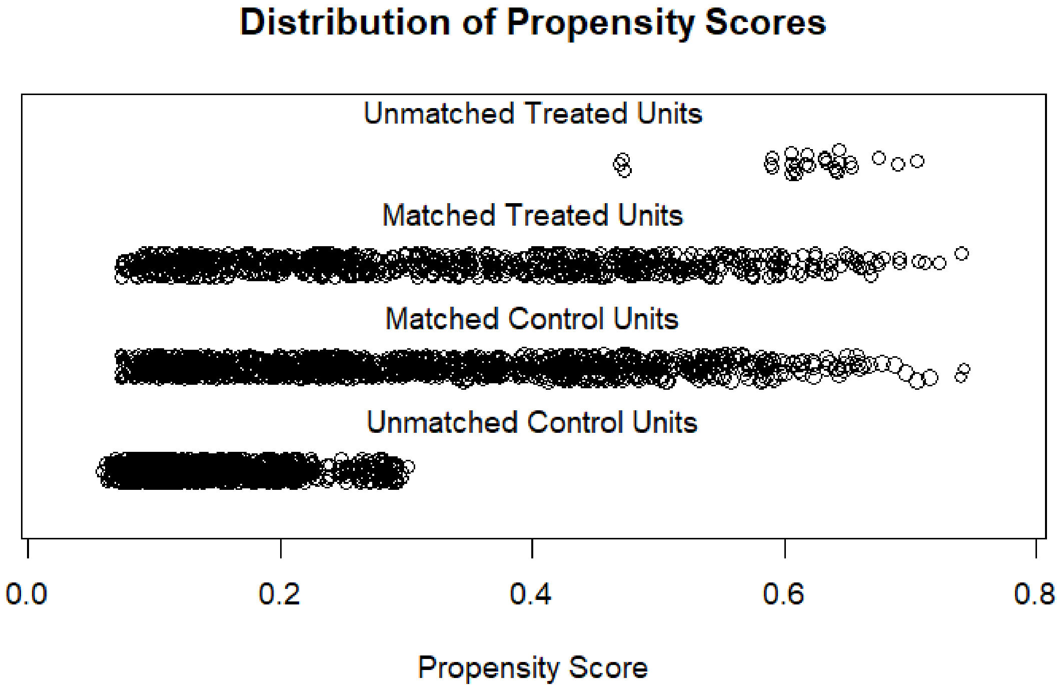

Supplemental Figure 2: Distribution of the propensity scores for matched and unmatched children in the sample for examining the effect of the intervention on BMI at age 10 years

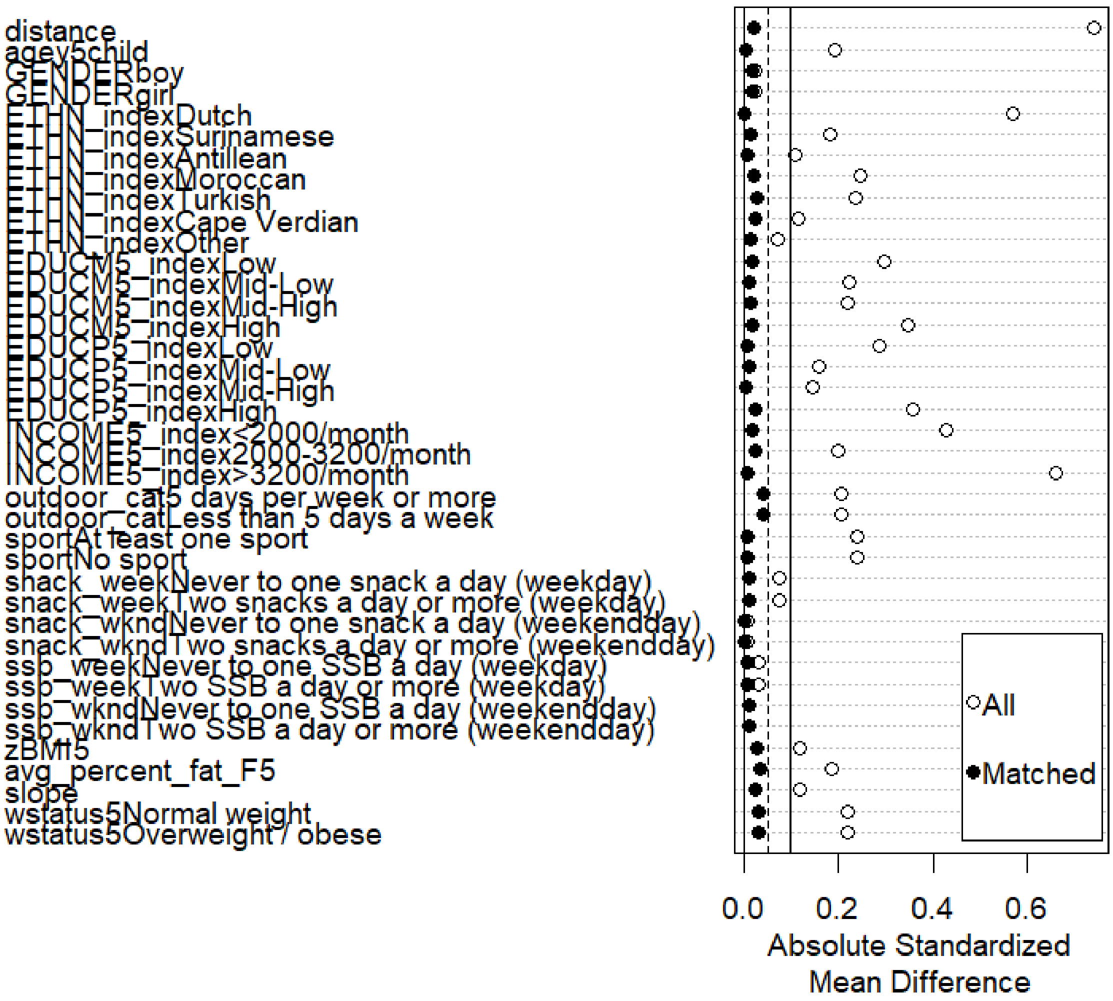

Supplemental Figure 3: Absolute standardized mean difference for baseline covariates in the original and the matched sample for examining the effect of the intervention on BMI at age 10 year

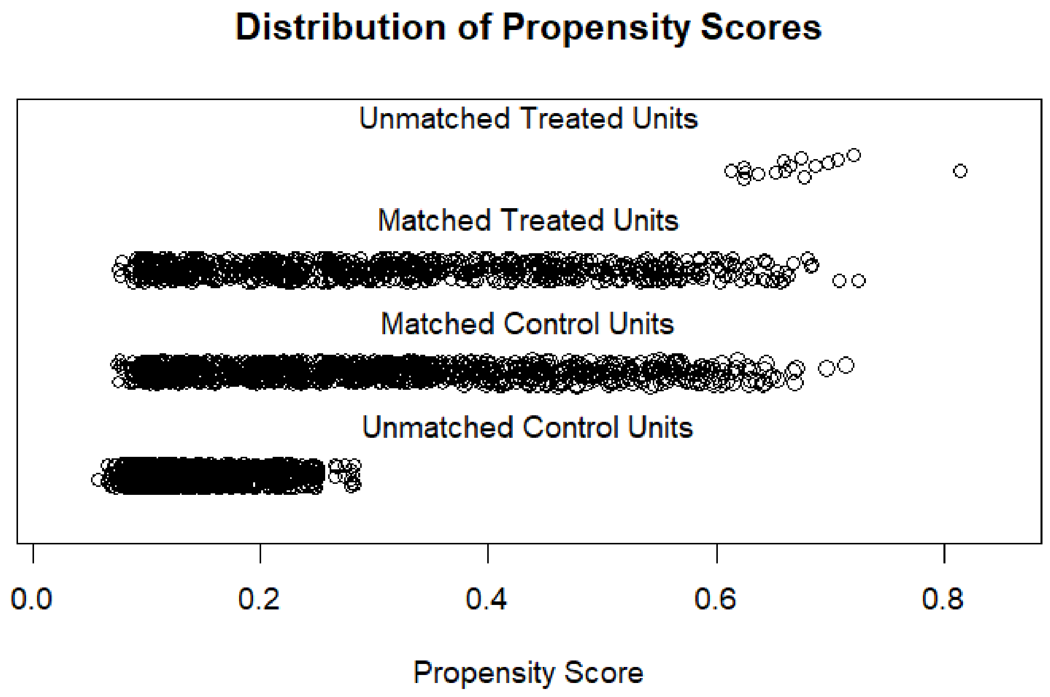

Supplemental Figure 4: Distribution of the propensity scores for matched and unmatched children in the sample for examining the effect of the intervention on BMI at age 14 years

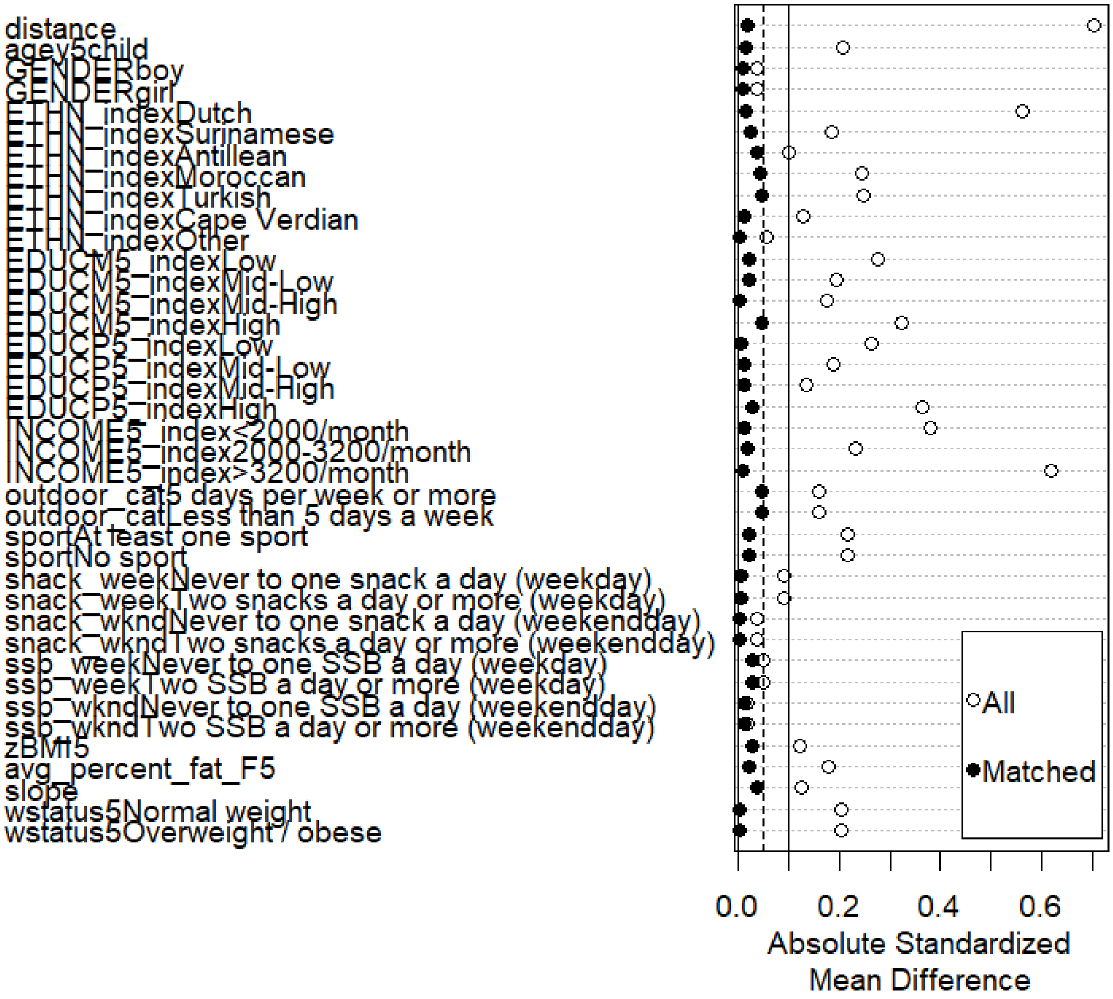

Supplemental Figure 5: Absolute standardized mean difference for baseline covariates in the original and the matched sample for examining the effect of the intervention on BMI at age 14 year

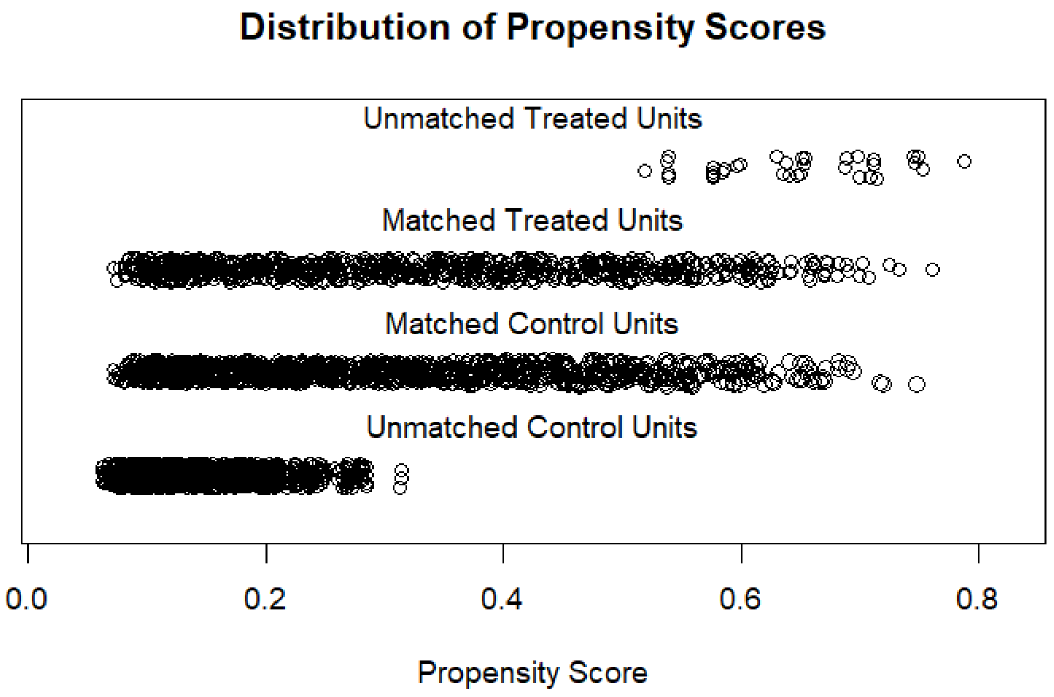

Supplemental Figure 6: Distribution of the propensity scores for matched and unmatched children in the sample for examining the effect of the intervention on fat mass at age 10 years

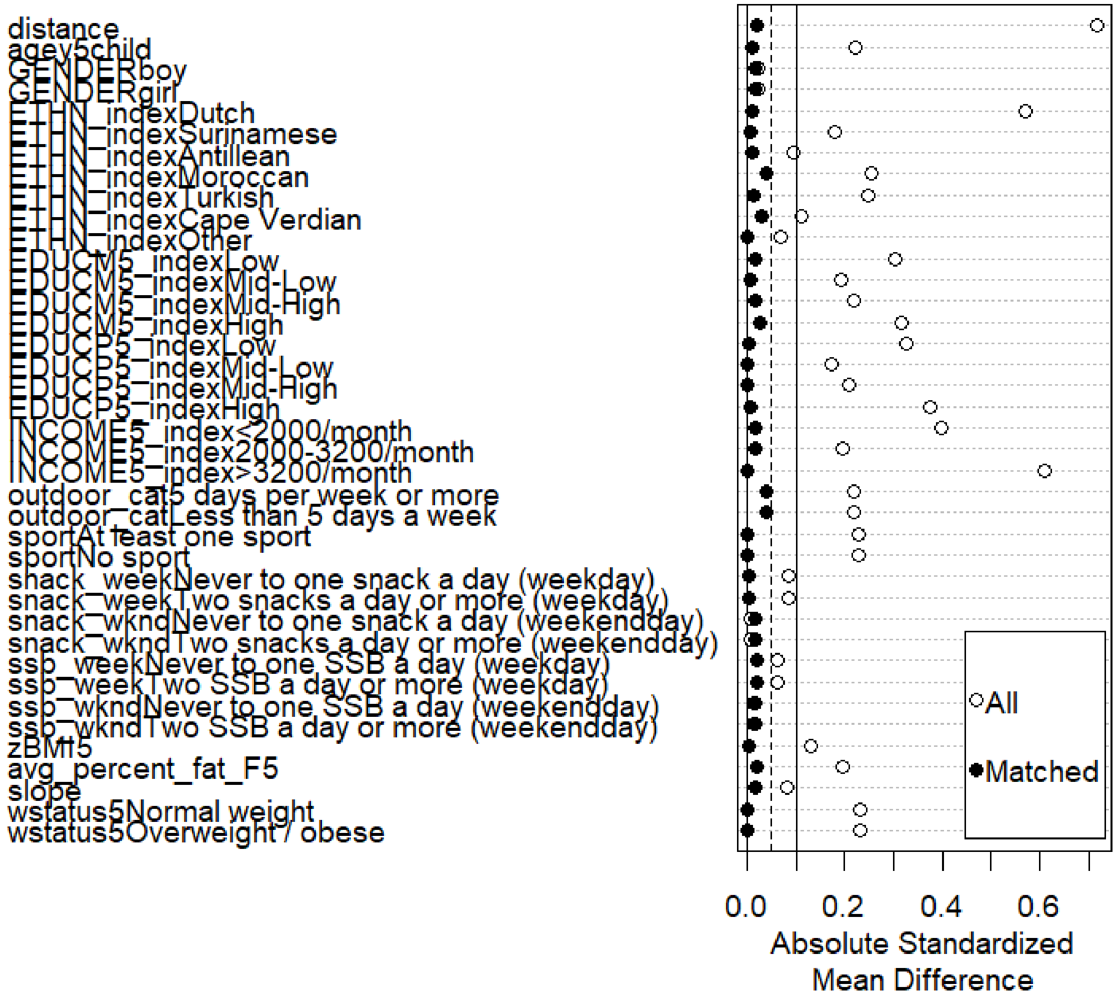

Supplemental Figure 7: Absolute standardized mean difference for baseline covariates in the original and the matched sample for examining the effect of the intervention on fat mass at age 10 year

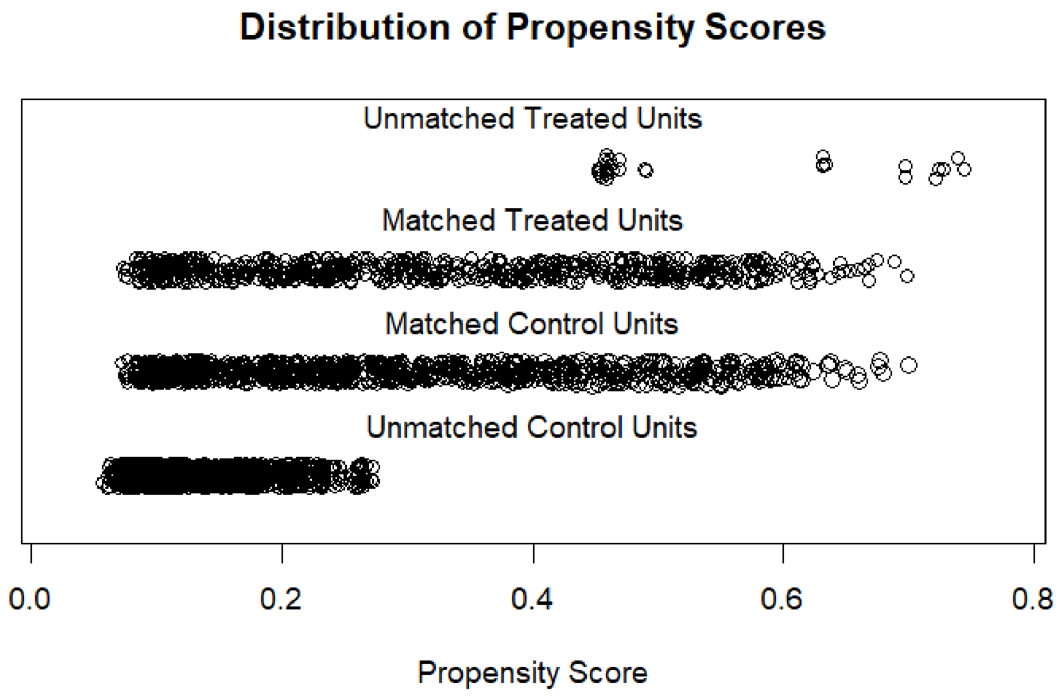

Supplemental Figure 8: Distribution of the propensity scores for matched and unmatched children in the sample for examining the effect of the intervention on fat mass at age 14 years

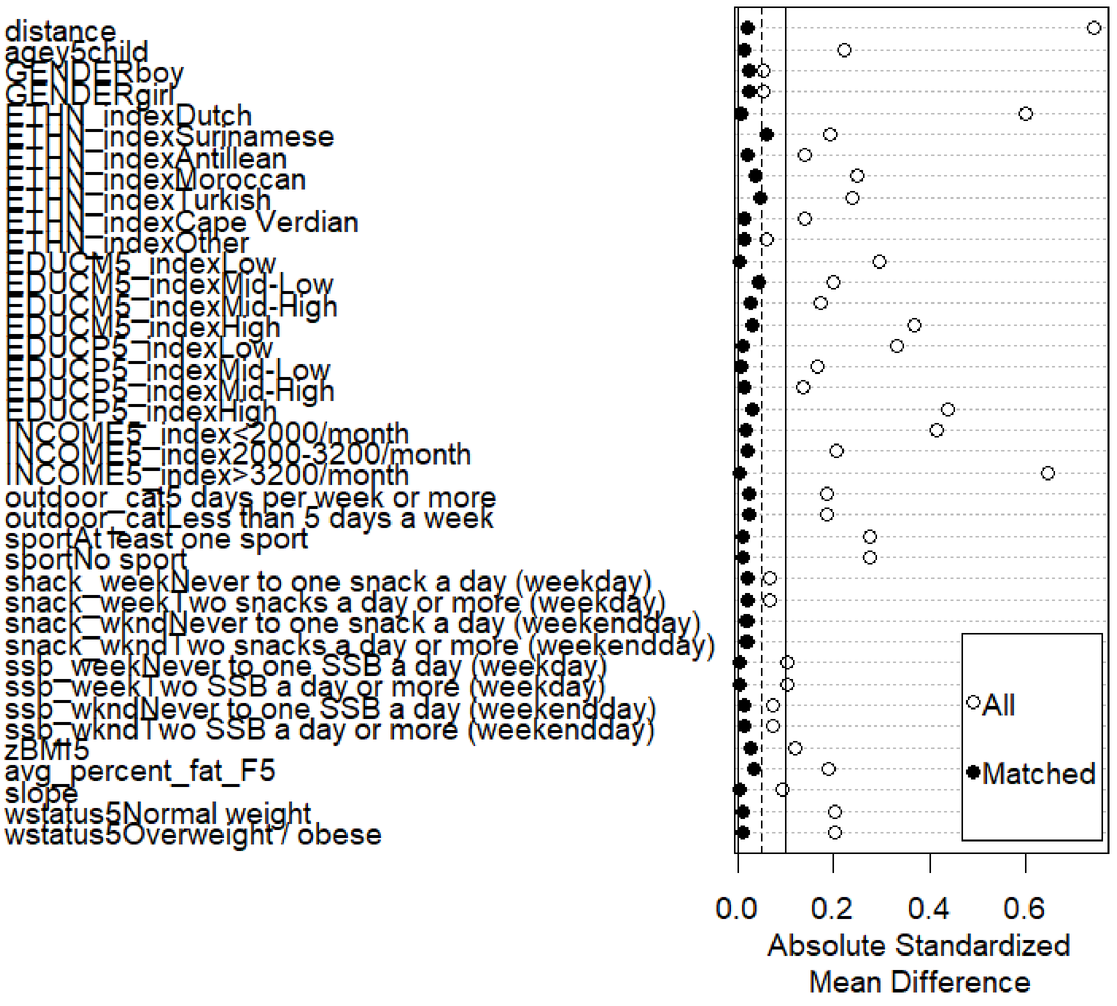

Supplemental Figure 9: Absolute standardized mean difference for baseline covariates in the original and the matched sample for examining the effect of the intervention on fat mass at age 14 year
